# Liver sinusoidal endothelial cell modulation upon resection and shear stress *in vitro*

**DOI:** 10.1186/1476-5926-3-7

**Published:** 2004-09-01

**Authors:** Filip Braet, Maria Shleper, Melia Paizi, Sergey Brodsky, Natalia Kopeiko, Nitzan Resnick, Gadi Spira

**Affiliations:** 1 Molecular Cell Biology Unit, Department for Molecular Biomedical Research, Ghent University / VIB, Technologiepark 927, B-9052 Ghent, Belgium; 2 Department of Anatomy and Cell Biology, The Bruce Rappaport Faculty of Medicine, Rappaport Family Institute for Research in the Medical Sciences, Technion, Haifa 31096, Israel; 3 Department of Physiology, The Bruce Rappaport Faculty of Medicine, Rappaport Family Institute for Research in the Medical Sciences, Technion, Haifa 31096, Israel; 4 Department of Medicine – Renal Research Institute, New York Medical College, Valhalla, NY 10595, USA; 5 Department of Experimental Surgery, The Bruce Rappaport Faculty of Medicine, Rappaport Family Institute for Research in the Medical Sciences, Technion, Haifa 31096, Israel; 6 Vascular Research Center, Department of Pathology, Brigham and Women's Hospital and Harvard Medical School, 11 Louis Pasteur Ave. NRB, Boston, MA 02115, USA

## Abstract

**Background:**

Shear stress forces acting on liver sinusoidal endothelial cells following resection have been noted as a possible trigger in the early stages of hepatic regeneration. Thus, the morphology and gene expression of endothelial cells following partial hepatectomy or shear stress *in vitro *was studied.

**Results:**

Following partial hepatectomy blood flow-to-liver mass ratio reached maximal values 24 hrs post resection. Concomitantly, large fenestrae (gaps) were noted. Exposure of liver sinusoidal endothelial cells, *in vitro*, to physiological laminar shear stress forces was associated with translocation of vascular endothelial cell growth factor receptor-2 (VEGFR-2) and neuropilin-1 from perinuclear and faint cytoplasmic distribution to plasma membrane and cytoskeletal localization. Under these conditions, VEGFR-2 co-stains with VE-cadherin. Unlike VEGFR-2, the nuclear localization of VEGFR-1 was not affected by shear stress. Quantification of the above receptors showed a significant increase in VEGFR-1, VEGFR-2 and neuropilin-1 mRNA following shear stress.

**Conclusion:**

Our data suggest a possible relation between elevated blood flow associated with partial hepatectomy and the early events occurring thereby.

## Background

Following partial hepatectomy (PHx) the remaining liver is transfused by normal blood volume, thereby exposing liver sinusoidal endothelial cells (LECs) to excess hemodynamic forces. These forces have been noted as an early event leading to liver restoration in rats [1-3]; however, the idea that quality of the blood rather than quantity has been the accepted dogma [4,5]. Based on time-scale events, shear stress inflicted on liver cells precedes the expression of factors some of which are expressed within minutes. Studies conducted in recent years indicate that shear stress induced NO leads to the expression of genes participating in liver regeneration including c-fos [6-8]. There is evidence demonstrating that increase of c-fos in PHx or portal branch ligation models is inhibited by N-nitro-L-arginine methyl ester, which blocks NO synthase [8]. The present study was undertaken to examine the molecular and ultrastructural effects of hemodynamic forces on LECs. We have chosen to focus on vascular endothelial cell growth factor (VEGF) receptors (VEGFRs), as these are present on endothelial cells and have been demonstrated not only to have a role in liver regeneration, but also to be affected by shear stress conditions. Following PHx [9], VEGF is expressed in periportal regions demonstrating lobular heterogeneity [10,11]. VEGFR-1 and VEGFR-2, as well as Tie 1, Tie 2 and platelet-derived growth factor, are all shown to increase in endothelial cells following PHx [12]. We have demonstrated the stimulatory effects of both VEGF-165 and VEGF-121 on liver cell proliferation following PHx [13,14]. In a recent study [15], it was shown that shear stress causes the induction and translocation of VEGFR-2 to the nucleus in bovine aortic endothelial cells. In addition, it promotes the formation of a complex comprising VEGFR-2, VE-cadherin and β-catenin. It is postulated that the complex acts as a shear stress receptor, mediating signals into the cells. Here, we describe the relationship between elevated blood flow to the liver following PHx and the morphology alterations associated with lining endothelial cells. We also provide evidence demonstrating that shear stress imposed on LECs *in vitro *is accompanied by a significant increases in VEGFR-1, VEGFR-2 and neuropilin-1 mRNA levels. Furthermore, following shear stress both receptors alternate from perinuclear and faint cytoplamic orientation to adhere to cytoskeletal components and cell membrane. These changes coincide with the behavior of the adherence junction proteins VE-cadherin and β-catenin.

## Results

### Portal blood flow following liver hepatectomy

**Figure 1:**
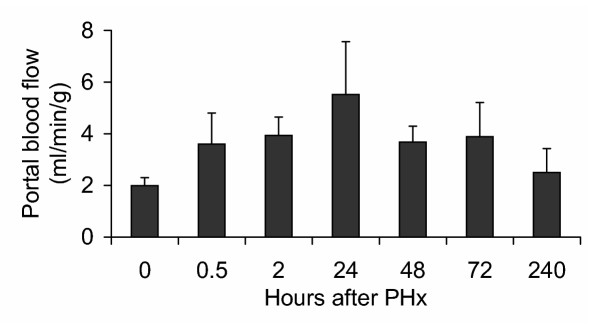
**Portal blood flow in normal and 70% partially hepatectomized rats. **At the designated time following partial hepatectomy rats were anesthetized and placed on a temperature controlled table. Following tracheotomy and saline infusion an ultrasound sensor was fixed to the portal vein. Blood flow was monitored by ultrasonic flowmetry. Results represent an average of 5 rats + 2 × SD.

Seventy percent of PHx is associated with cell proliferation and a gradual increase in liver mass (data not shown). Nine days post-hepatectomy close to 80% of the original liver weight was restored. PCNA labeling index peaked at 48 hrs thereby returning to preoperative values. Concomitant with liver resection an immediate increase in blood flow to the remnant liver was evident, reaching a maximum of 2.5 fold at 24 hrs (Fig. 1). Elevated values remained for as long as 72 hrs. Ten days following partial hepatectomy blood flow returned to normal. Values recorded earlier than 20 minutes are subject to technical difficulties; therefore, they are not presented.

### LEC Ultrastructure following partial hepatectomy

The effects of partial hepatectomy and the associated shear stress developing as a result of excessive blood flow to the remnant liver were evaluated with the aid of scanning electron microscopy. Special emphasis was given to the influence of these forces on the surface of liver sinusoids and intactness of the endothelial lining.

**Figure 2:**
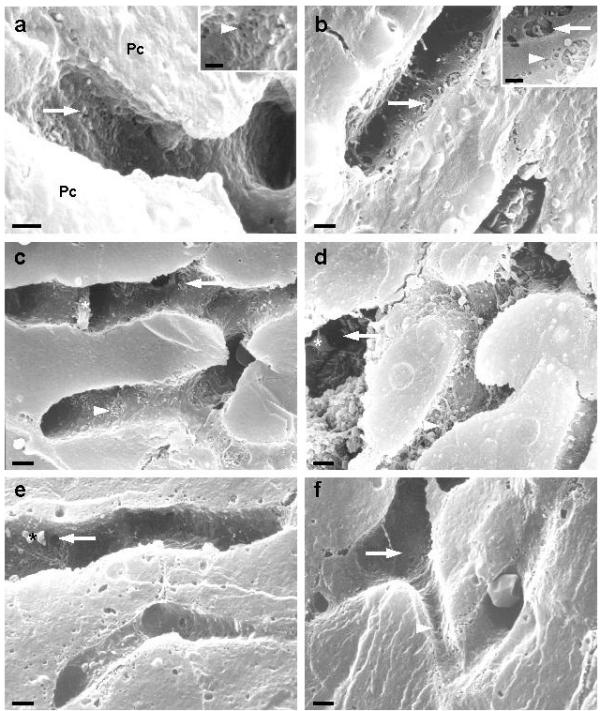
**Scanning electron micrographs of liver periportal sinusoids. **(a) control, (b-f) following partial hepatectomy; control liver (a) demonstrates an intact fenestrated wall (arrow) and undisrupted bordering parenchymal cells (Pc). Inset depicts fenestrae (arrowhead). (b) Numerous gaps (arrow) are observed as early as ten minutes after PHx. Inset shows a detailed image of gaps (arrow) and fenestrae (arrowhead). (c) 24 hrs after PHx, gaps (arrow) are still present. Note the protruding microvilli from the underlying parenchymal cell surface (arrowhead). Small structures (*), probably platelets, could be noticed adhering to endothelial wall. 72 hrs (d) and 168 hrs (e) after PHx, depicting features similar to those seen in (c). (f) Ten days after PHx an intact endothelial lining (arrow) and fenestrae (arrowhead) could be observed. Scale bars: 2 μm; Insets: 0.5 μm.

Under normal conditions, liver lobule sinusoids show an intact endothelial lining, consisting of LECs with flattened processes perforated by small fenestrae. These fenestrae measure 0.15–0.2 μm in diameter and are arranged in groups, sieve plates (Fig. 2a). As early as ten minutes post hepatectomy, endothelial changes were already noted in the form of fused fenestrae (gaps), ranging in size between 0.3 μm and 2 μm (Fig. 2b). These gaps were more prominent in periportal than pericentral areas (Table 1). Increasing values were noted in subsequent times, 24 (Fig. 2c), 72 (Fig. 2d) and 168 hrs (Fig. 2e) post-surgery in both areas. Ten days after hepatectomy, the morphology of the endothelial lining (Fig. 2f) and number of gaps returned to preoperative conditions (Table 1).

**Figure 3:**
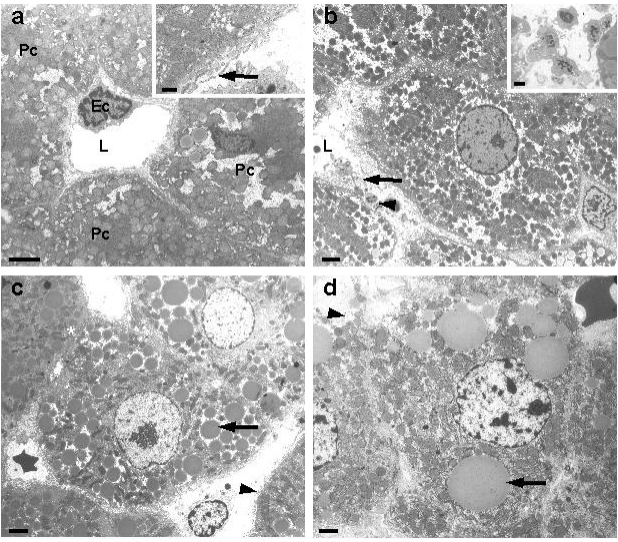
**Transmission electron micrographs of liver periportal areas. **(a), control, (b-d), after partial hepatectomy; (a) illustrates an intact histological relationship between liver sinusoidal endothelium (Ec) and neighboring liver parenchymal cells (Pc). Note the patent lumen (L). Inset depicts the intact cytoplasmic processes of endothelial cells bearing fenestrae (arrow). (b) Ten minutes after PHx, the surface area of the sinusoidal lumen (L) decreases and severe damage of the endothelial lining in the form of gaps is noted (arrow). Blood platelets (arrowhead) adhere to the damaged sinusoidal lumen. Inset shows a detailed image of blood platelets. (c) 24 hrs after PHx. Fat droplets (arrow) are evident in the cytoplasm of parenchymal cells. Gaps are still present (arrowhead). (d) 72 hrs after PHx reveals endothelial damage (arrowhead) and large fat droplets (arrow) within the parenchymal cells (compare with Figure 3c for the difference). Scale bars: 2 μm; Insets: 0.5 μm.

Transmission electron microscopy was used to study in great detail the above alterations. (Fig. 3). Control tissue showed an intact relationship between LECs and neighboring liver parenchymal cells (Fig. 3a). The sinusoid was patent and empty; the wall of the sinusoid was composed of a thin layer of fenestrated endothelium covering the space of Disse, filled by microvilli extending from the parenchymal cell surface. These parenchymal cells contained glycogen, a few lipid vesicles, and numerous organelles in their cytoplasm (Fig. 3a). Ten minutes after hepatectomy, many blood platelets adhered to the endothelial lining. In addition, the endothelial lining became disrupted as represented by the occurrence of gaps and microvilli, which were facing directly toward the sinusoidal lumen (Fig. 3b). These morphological alterations were still present 24, 72 and 168 hrs after PHx. Lipid accumulation in the form of droplets could be observed in the cytoplasm of parenchymal cells 10 hrs (data not shown) after partial hepatectomy, persisting until day 3 (Fig. 3d). To avoid any possible effect caused by the procedure and anesthetic reagents used, a sham operation was conducted (time 0).

### Effect of laminar shear stress on the expression and distribution of VEGF receptors in liver endothelial cells

**Figure 4:**
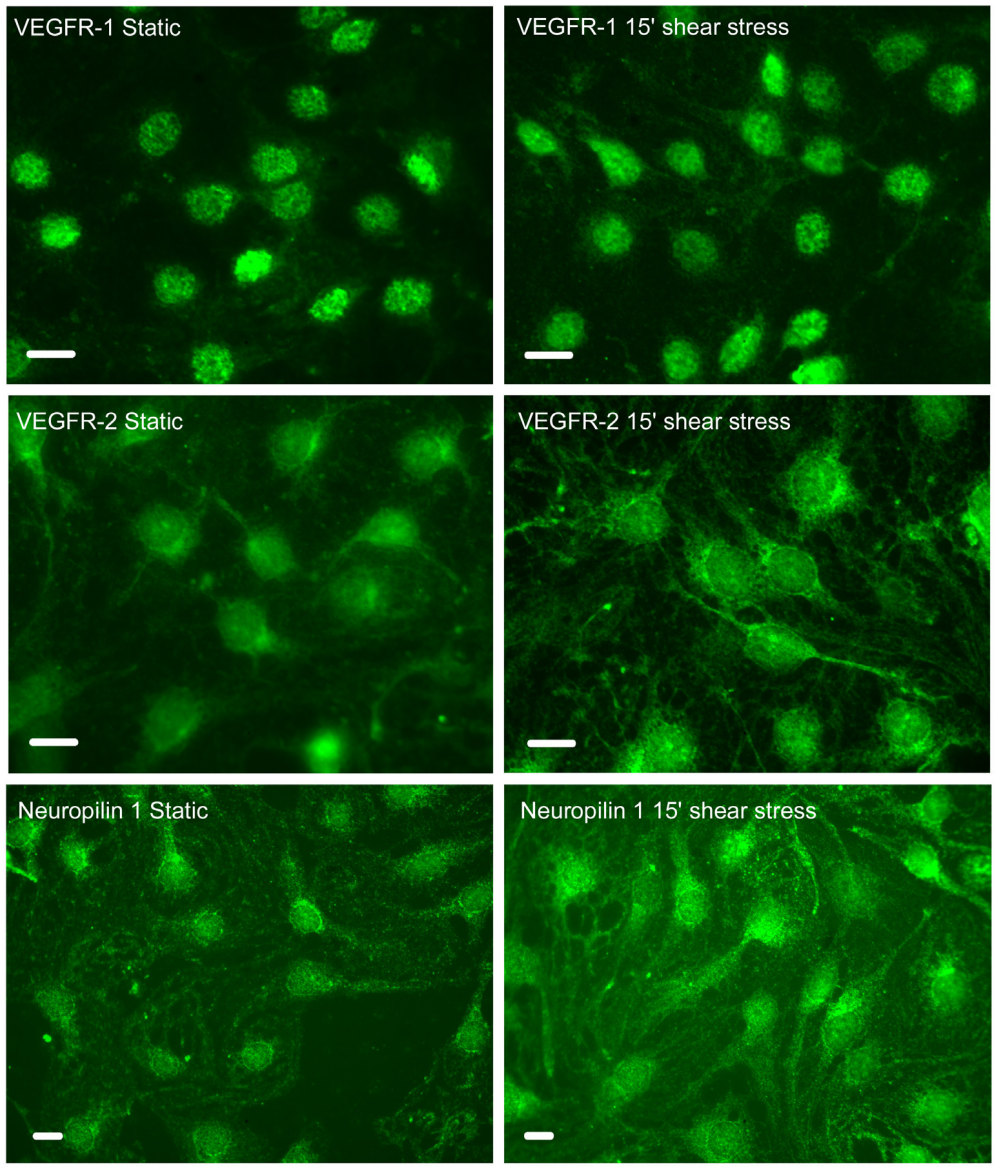
**Immunofluoresence of LECs before and after shear stress. **LECs were reacted with anti VEGFR-1, VEGFR-2 and neuropilin-1 before and after exposure to laminar shear forces (10 dynes/cm^2^/15 minutes). Cy2 conjugated labeled second antibodies were used to visualize the binding of the appropriate antibody. Scale bars: 2 μm.

Purified LECs grown in culture retained their characteristic sieve plates (data not shown). Following 4 hrs of incubation at 37°C and extensive washing, the cells demonstrated nuclear localization of NFκb suggesting an active state. To avoid activation, purified LECs were grown in feeding medium containing 0.25% FCS for 4 hrs, extensively washed and left for 12 hrs before further used. Under these conditions, more than 94% of the cells exhibited cytoplasmic NFκb which was re-localized to the nucleus following shear stress (data not shown). LECs displayed perinuclear and cytoplasmic localization of VEGFR-2 and neuropilin 1 (Fig. 4). Following exposure to shear stress conditions (10 dynes/cm^2^/15 minutes), a strong cytoplasmic presence was evident, with a clear tendency to adhere to cytoskeletal components. VEGFR-1 displayed nuclear localization, which was unchanged when shear stress was applied.

**Figure 5:**
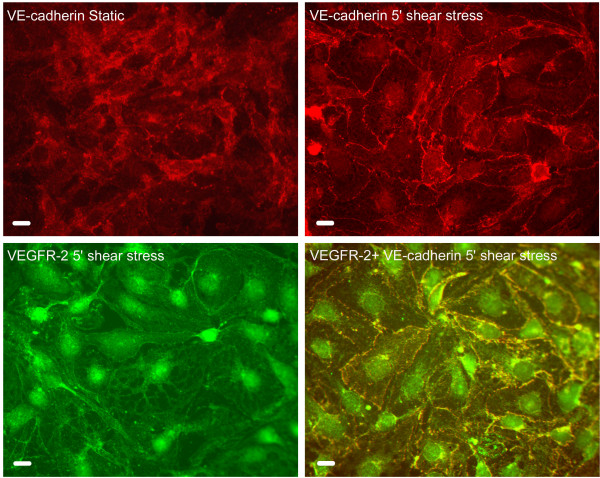
**Immunofluoresence of LECs before and after shear stress. **LECs were reacted with anti VE-cadherin and VEGFR-2 alone and in conjunction before and after exposure to laminar shear forces (10 dynes/cm^2^/5 minutes). Cy2 and rhodamine (TRITC) conjugated labeled second antibodies were used to visualize the binding of the appropriate antibody. Scale bars: 2 μm.

Owing to the tendency of VE-cadherin and β-catenin to react with cytoskeletal proteins under hemodynamic forces, both were followed in LECs under static conditions and shear stress. Co-staining analysis of both suggests the formation of a complex demonstrating a strong tendency to the membrane (data not shown). Co-staining of VE-cadherin and VEGFR-2 (Fig. 5) exhibits similar profile, pointing to the existence of a possible complex, composed of the two proteins.

**Figure 6:**
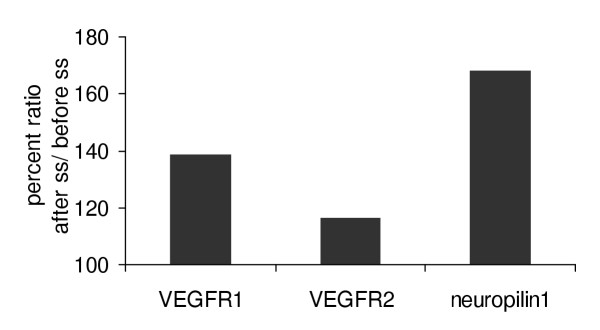
**Real time PCR of VEGFR-1, VEGFR-2 and neuropilin-1 before and after exposure of LECs to laminar shear stress. **Pooled RNA from six different experiments was isolated from LECs subjected to laminar shear stress forces (10 dynes/cm^2^/15 minutes) and used to measure mRNA levels of the respective receptors.

Real time RT-PCR was used to quantify the amount of mRNA of all receptors before and after shear stress (Fig. 6). The results shown represent pooled RNA isolated from six animals. It is evident that VEGFR-1, VEGFR-2 and neuropilin-1 levels increase following shear stress conditions.

## Discussion

Liver regeneration is associated with an increased expression of a diverse number of genes including immediate early genes, delayed genes, cell cycle and DNA replication and mitosis genes [4,5]. Some of these genes increase within moments after PHx; others increase hours post-surgery. Regardless of the timeframe, the most obvious change occurring immediately after PHx is an elevated in hemodynamic forces imposed on liver cells. Those changes are the result of an increase in the ratio of blood flow to liver weight. We documented a 2.5 fold increase in portal blood flow following 70% PHx. These changes occur immediately and persist for a number of days.

Endothelial cells lining liver sinusoids are likely to be the first to sense changes in shear stress. Those cells are unique as they have no typical basal lamina. Moreover, the cells are fenestrated allowing free passage of chylomicrons, lipoproteins, hormones, growth factors and proteases [16]. The size and density of these fenestrae are affected by physical factors, such as portal pressure and shear stress, as well as soluble factors [17-20].

Exploring the effects of shear stress on LECs *in vivo *is, at the moment, beyond our reach. Therefore, the present study examines the effects of increased blood flow following PHx on the morphology of LECs. We also follow the gene expression and protein distribution in LECs exposed to controlled shear stress *in vitro*. These forces mimic to the best of our ability physiological conditions.

Following 70% PHx an immediate ultrastructural change was noted in the form of fused fenestrae and gaps. Their number increased significantly in both periportal and pericentral areas (Fig. 2); yet, expressed differently in both zones (Table 1). This observation is not surprising in light of other zonation gradients reported for many liver functions [16,21-23], like fenestration pattern, differential expression of receptors, hepatocyte metabolism, and ECM-distribution in the space of Disse. Different high-resolution microscopic methods have shown that gaps may originate from the fusion of several fenestrae [24,25]. In fact, gaps along the endothelial lining have been noted when different sample preparation methods were applied [16,24,26] or be induced by hepatotoxins [27] and high-perfusion pressure [28]. In accordance with our observation, Wack et al. [29] reported a gradient behavior in porosity between periportal and pericentral areas following 70% PHx, surprisingly though the gradient described by those authors persists only at 5 minutes and 24 hrs post-surgery. In this study, diameter determinations on gaps were omitted making full comparison difficult. In our experiments, we could not detect statistical variations in the size of gaps between the two zonal areas (our unpublished data). This could be explained by the fact that the size of gaps varied between 0.3 μm and 2 μm and mean values with large standard errors were obtained, excluding therefore valuable statistical analysis.

**Table 1:** Number of gaps along the sinusoidal endothelial lining following partial hepatectomy

**Time**	**Periportal (zone 1) n gaps / 10 μm**^2^	**Pericentral (zone 3) n gaps / 10 μm**^2^
Control	0.10 (0.14)	0.06 (0.12)
10 min	1.57 (0.74)*	0.33 (0.13)^§^
24 hrs	1.47 (0.66)*	0.47 (0.32)^§^
72 hrs	2.18 (0.91)*	0.84 (0.65)^§^
168 hrs	2.28 (0.88)*	0.79 (0.55)^§^
240 hrs	0.09 (0.05)	0.07 (0.05)

Morphometric analysis evaluating the number of gaps per area along the sinusoidal endothelial lining, studied by SEM. Results are expressed as mean (standard deviation) and significance was determined with the Mann Whitney two-sided U-test. The symbols * and ^§^denote significant differences between control and respective time points (p ≤ 0.05). Significant differences (p ≤ 0.05) between the number of gaps in the periportal and pericentral zones were also noted at all time points following partial hepatectomy except day 10 and control. For every group, n = 3.

In our experiments (Fig. 1), maximal values of blood flow per mg of liver were determined at 24 hrs thereby returning to baseline levels. The inconsistency between the number of gaps and the ratio of blood flow per mg of liver tissue, at later time, points may either reflect the time lapse required for liver tissue to recover or that portal pressure is not the only factor influencing lining endothelial cells. Consistent with the increased permeability in zone 1 and zone 2 following PHx, accumulation of lipid droplets was evident 10 hrs post surgery, persisting until day three. At the completion of liver regeneration, lipid content returns to normal values [18]. Increased lipid uptake seems to correlate with the change in barrier competence presented by sinusoidal endothelial cells; however, the role it has in the regenerating liver is still to be elucidated.

Given the increase in blood flow to the liver immediately after PHx, it is likely that the "damage" caused to LECs is the result of excessive shear stress to which the cells are exposed. Interestingly, injections of large volume at a short time, hydrodynamic injections [30] inflict periportal and pericentral damage in the form of large (fused) fenestra (our data to be published).

Shear stress conditions can artificially be applied using the cone and plate apparatus [31]. We have chosen to limit our observation to VEGF receptors as those were shown to be expressed on endothelial cells and their level changed during liver regeneration. Owing to the fact that neuropilin-1 acts as VEGF co-receptor, we have looked at neuropilin-1 expression following shear stress as well.

LECs exhibited nuclear staining of VEGFR-1. This localization was not affected by shear stress conditions. VEGFR-2 and neuropilin-1 present a similar pattern of perinuclear and faint cytoplasmic presence. Following shear stress conditions the two receptors seemed to adhere to membrane and cytoskeletal components.

Neuropilin-1 is an isoform specific receptor for VEGF-165 [32], VEGF-E [33], PLGF152 [34] and VEGF-B [35]. Recent studies have demonstrated a complex dependent signaling involving VEGF-165, neuropilin-1 and VEGFR-2 [36]. Such a complex was shown to exist on the surface of endothelial cells or between tumor cells and endothelial cells. Activation of VEGFR-2 has been shown to be involved in the formation of complexes with various cytoplasmic proteins including adherence junction proteins [37,38]. Furthermore, nuclear translocation of VEGFR-2 along with caveolin-1 and eNOS was reported to occur following VEGF treatment [39]. Consistent with data recently presented [15], VEGFR-2 co-stains with VE-cadherin following exposure to shear stress. Our preliminary data point to the possibility of a large complex consisting of VEGFR-2, neuropilin-1 and the adherence junction proteins VE-cadherin and β-catenin; nonetheless, additional experiments need to be done before any conclusion can be reached. Coinciding with the intense staining of the above following exposure to shear stress are the increased mRNA levels of all three as detected by real time PCR.

Hemodynamic forces play a major role in restructuring blood vessels by modulating endothelial structure and functions such as increased permeability to macromolecules or damage to endothelial cells [40]. Therefore, a key question in liver regeneration is how these forces imposed during the early steps following resection are translated into gene expression, DNA synthesis and cell proliferation. Shear forces dependent signaling is presumably based on cytoskeletal components, which act as a mechano-transducer. Indeed, tyrosine phosphorylation of the endothelial cell adhesion molecule PECAM-1 is observed in response to flow [40].

## Conclusions

In summary, the present study documents an increase in blood flow to remnant liver following PHx. This change is associated with an elevated number of endothelial cell gaps in both periportal and pericentral areas. Shear stress *in vitro *induces in endothelial cells membrane translocation of VEGFR-2 and neuropilin-1. It is conceivable that under shear stress conditions a complex consisting of VEGFR-2/neuropilin-1 and adhesion molecules forms. Such a complex may well be formed following the elevated blood flow associated with partial hepatectomy, playing a role in the early signals leading to liver regeneration.

## Methods

### Animals and surgical procedures

Male Sprague-Dawley rats weighing 300–325 g were used. PHx was performed on 5 animals under light anesthesia by removing the right lateral and median lobes[41]. At different time intervals animals were exsanguinated, the liver removed and tissue samples were prepared for immunostaining and RNA extraction. Animals undergoing PHx and analyzed by electron microscopy were anesthetized first by Ketamine and Xylasine followed by intubation with isoflurane 1.5%. Animals received humane care according to the criteria outlined in the "Guide for the care and use of laboratory animals" NIH publication.

### Monitoring liver regeneration

Liver regeneration was monitored using liver mass and PCNA. Liver mass was calculated by weighing the removed lobes following surgery and the regenerating liver at the indicated time point. For PCNA immunostaining, specimens were fixed in paraformaldehyde, embedded in paraffin and sliced. Sections were incubated with anti-PCNA followed by biotin conjugated secondary antibody. The binding of anti-PCNA was monitored using avidin-peroxidase and amino ethyl carbazol as a substrate (Zymed, San Francisco, CA).

### Blood flow

Five rats were anesthetized and placed on a temperature-controlled table. Following tracheotomy and saline infusion an ultrasound sensor was fixed to the portal vein. Portal blood flow was monitored by ultrasound flowmetry and automatically recorded (Ultrasonic System Inc. model T206, Ithaca, N.Y).

### Preparation of liver tissue for electron microscopy

Tissue samples were prepared according to standard protocols [16]. Briefly, samples were cut into 1 mm^3 ^blocks in 1.5% glutaraldehyde, in 0.12 M sodium cacodylate buffer. Following fixation, blocks were submerged in 1% osmium tetroxide, dehydrated in ethanol and embedded in Epon. Semithin (1 μm) sections were cut and stained with 1% toluidine blue solution. For detailed EM-study, 50–80 nm ultrathin sections were stained first with uranyl acetate and then with lead citrate. For SEM, dehydrated blocks were dried with hexamethyldisilazane and subsequently broken in liquid nitrogen, mounted on stubs and sputter coated with a thin layer of 20 nm gold [24]. Morphometric analysis was performed on randomly acquired digitized SEM images at magnifications ×5,000 or ×20,000, as previously described [42]. The UTHSCSA Image Tool 2.0 software was used to determine the number of liver sinusoidal endothelial gaps. Gaps, an empty area, a hole with a maximum diameter of ≤ 0.3 μm and ≤ 2 μm, were discriminated from fenestrae based on morphology and size [16,24,27]. For each experimental variable, 10 images in the periportal and pericentral zones (regions up to 100 μm in diameter) were randomly selected and captured at both magnifications. Three animals were tested at each time point. All experiments were repeated three times and data were expressed as mean (plus standard deviation of the mean).

### Isolation of liver endothelial cells (LECs)

LECs were isolated using a modification of the procedure described by Braet et al. [42] and Smedsrod and Pertoft [43]. Briefly, the liver was washed and perfused through the portal vein with balanced salt solution and 0.05% collagenase A. Following excision and mincing, the cells were filtered and centrifuged. Enriched liver sinusoidal cells were then layered on a two-step percoll gradient (25/50%) and centrifuged for 20 minutes at 900 g. The intermediate, 25/50% zone is enriched with LECs and Kupffer cells. Following selective adherence of Kupffer cells, LECs were spread on collagen coated plastic slides for 4 hrs and extensively washed. Based on EM such cultures are estimated to be 95% pure.

### *In vitro *shear stress

LECs grown on plastic collagen-coated slides were subjected to shear stress forces produced between a stationary base plate and a rotating cone [31]. High-level shear stress forces of 10 dynes/cm^2 ^were enforced for 5 or 15 minutes at which time the cells were washed and either used for immunofluorescence or RNA extraction.

### Immunofluorescence

Cells were fixed in 2% paraformaldehyde followed by 1% triton paraformaldehyde solution. The slides were then immersed in blocking solution and stained with either anti VEGFR-1, VEGFR-2, neuropilin-1, VE-cadherin, β-catenin or NFκb. Cy2 or rhodamine (TRITC) conjugated secondary antibodies were used.

### RNA extraction

RNA was extracted from LECs by the RNAeasy kit (Qiagen, Chatsworth, CA) according to manufacturer's protocol and treated with DNase.

### Real time RT-PCR

RNA samples were reversed transcribed and amplified using the QuantiTect SYBR Green RT-PCR kit (Qiagen) and appropriate primers at concentrations of 90 nM to 125 nM. The one-step RT-PCR was carried out at a Rotor-Gene 2000 real time cycler (Corbett Research, Australia). The thermal cycling conditions included 95°C for 15' followed by 45 cycles of amplification at 94°C 20", 60°C 15–30", 72°C 15". Samples were monitored after elongation by SYBR Green dye binding to the amplified double stranded DNA at 72°C–78°C. All samples were amplified in duplicates and each experiment was repeated twice. Quantitation was carried out using a standard curve. The Rotor-Gene analysis software was used for the calculation of the amount of each RNA sample.

### Statistical analysis

Significance was determined with the Mann Whitney two-sided U-test. Differences were considered significant when when p ≤ 0.05.

## Authors' contributions

FB and GS conceived the design and coordination of the study and drafted the manuscript and assessed LEC ultrastructure, MS carried out cell isolation, shear stress experiments and immunofluorescence. MP carried out Real time PCR and participated in animal procedures and drafting the paper. SB carried out portal blood flow evaluation. NK participated in animal procedures, NR participated in design and coordination. All authors read and approved the final manuscript.

## Acknowledgments

Israel Science Foundation 537/01, Chief Scientist's Office of the Israel Ministry of Health 5002, Mars-Pittsburgh Foundation for Medical Research 182-012, Rappaport Family Institute Fund. This research was partially supported by the "Fund for Scientific Research-Flanders" (grant N° 1.5.001.04N (F.B.)). F.B. is a postdoctoral researcher of the "Fund for Scientific Research-Flanders".
